# Antimicrobial resistance profiles of and associated risk factors for *Pseudomonas aeruginosa* nosocomial infection among patients at two tertiary healthcare facilities in Lusaka and Copperbelt Provinces, Zambia

**DOI:** 10.1093/jacamr/dlae139

**Published:** 2024-09-16

**Authors:** Patrice Ntanda Mukomena, Martin Simuunza, Sody Munsaka, Geoffrey Kwenda, Flavien Bumbangi, Kaunda Yamba, Josephine Kabwe, Jean-Marie Kayembe, John Bwalya Muma

**Affiliations:** Department of Medicine, School of Medicine, Eden University, Lusaka, Zambia; Department of Disease Control, School of Veterinary Medicine, University of Zambia, Lusaka, Zambia; Department of Disease Control, School of Veterinary Medicine, University of Zambia, Lusaka, Zambia; Department of Biomedical Sciences, School of Health Sciences, University of Zambia, Lusaka, Zambia; Department of Biomedical Sciences, School of Health Sciences, University of Zambia, Lusaka, Zambia; Department of Medicine, School of Medicine, Eden University, Lusaka, Zambia; Department of Disease Control, School of Veterinary Medicine, University of Zambia, Lusaka, Zambia; Department of Disease Control, School of Veterinary Medicine, University of Zambia, Lusaka, Zambia; Department of Medicine, School of Medicine, Eden University, Lusaka, Zambia; Department of Medicine, School of Medicine, University of Kinshasa, Kinshasa, Democratic Republic of Congo; Department of Disease Control, School of Veterinary Medicine, University of Zambia, Lusaka, Zambia

## Abstract

**Background:**

Antimicrobial resistance (AMR) of pathogens such as *Pseudomonas aeruginosa* is among the top 10 threats to global health. However, clinical and molecular data are scarce in Zambia. We, therefore, evaluated the AMR profiles of *P. aeruginosa* nosocomial infections (NIs).

**Methods:**

A year-long hospital-based cross-sectional study was conducted at two large tertiary-level hospitals in Zambia. Patients with current or previous hospital contact were screened for NIs. The current study focused on patients diagnosed with *P. aeruginosa* NIs. Clinical specimens were collected for bacteriological culture, and PCR amplification of 16S rRNA gene fragments was performed on pure isolates. Hospital or NIs were defined as infections that arise during hospitalization, occurring at least 48 h after admission. The Kirby–Bauer’s disk diffusion method was used to evaluate antibiotic resistance patterns. The association between AMR and risk factors was analysed using the χ^2^ test.

**Results:**

Eight hundred and forty-one patients were screened, and clinical specimens were collected and analysed. Of them, 116 (13.7%) were diagnosed with *P. aeruginosa* NIs. The participants’ ages ranged from 15 to 98 years, with a mean of 51 (SD ± 18). Catheter-associated urinary tract infections (57%) were the most common, followed by pressure sores (38.7%). *P. aeruginosa* isolates were primarily susceptible to amikacin, which had the highest resistance to FEP. We observed a high prevalence of multidrug resistance (73.6%). The AMR was associated with carbapenem-hydrolysing β-lactamase gene blaOXA-51 and surgical care.

**Conclusions:**

This study has demonstrated that multidrug-resistant *P. aeruginosa* is prevalent in hospitals in Zambia’s Lusaka and Ndola districts and possibly countrywide.

## Introduction

The discovery of PEN at St Mary’s Hospital in London in 1928 by Alexander Fleming was a breakthrough in medical science and practice. This discovery led to the introduction of antibiotics, significantly reducing the number of infection-related deaths. Therefore, predictions were made that infectious diseases would finally be eliminated as more antimicrobial compounds were discovered.^1^ Unfortunately, developing and rising antimicrobial resistance (AMR) to these antibacterial agents quickly diminished this optimism.^2^ Since then, the WHO has declared AMR as one of the top 10 global public health threats facing humanity.^3^ It is estimated that AMR will lead to 10 million annual human deaths globally by 2050 if no interventions are implemented to combat it at global, regional and national levels.^4^ Despite scanty information in Africa, the available data show evidence of increasing trends in AMR, suggesting that the region shares the worldwide trend of this problem.^5^

Gram-negative bacteria, especially non-fermenting pathogens, have been implicated in nosocomial infections (NIs). *Pseudomonas aeruginosa*, one of the most common hospital pathogens, is a causative agent of many severe opportunistic infections, particularly in immunocompromised patients.^6^ High morbidity and mortality occur due to weakened host defences, bacterial resistance to antibiotics and the production of extracellular enzymes and toxins.^7^ β-Lactamase production has significantly contributed to the rise in resistance to beta-lactam antibiotics, becoming a public health threat.^8^ The emergence and the spread of metallo-β-lactamases, especially in non-fermenters like *P. aeruginosa*, have become therapeutic challenges. Class C β-lactamases (Amp C) confer resistance to the same antibiotics as ESBLs with additional resistance to cephamycin and β-lactamase inhibitors.^9^ Infections caused by AMR bacteria pose serious challenges that include prolonged hospital stays, higher hospital costs and poorer clinical outcomes in that they cause severe morbidities and mortality.^9,10^

Due to the need for country-specific data on the burden of AMR and the factors driving its spread, the Zambia National Public Health Institute recently developed an integrated AMR surveillance framework to guide the fight against AMR.^11^ A recent study by Kaluba *et al*.^12^ found low carbapenem resistance in *P. aeruginosa*. It recommended improved AMR surveillance, antimicrobial stewardship and infection prevention and control at the University Teaching Hospital (UTH) in Zambia. Nevertheless, specific clinical and molecular AMR data on resistant *P. aeruginosa*, such as data on associated genes, are scarce.^11,12^ Therefore, this study evaluated antimicrobial and molecular profiles of *P. aeruginosa* from patients with NIs at two sizeable tertiary healthcare facilities in Zambia.

## Materials and methods

### Study site and design

A hospital-based cross-sectional study was conducted between April 2020 and April 2021 at the UTH in Lusaka district and the Ndola Teaching Hospital (NTH) in Ndola district, Zambia. The UTH is a 2000-bed capacity tertiary-level hospital and the largest referral centre in Zambia. At the same time, the NTH has a capacity of 948 beds and is the second largest hospital in the country after the UTH. The UTH is located in the Lusaka district, the capital city of Zambia, and serves as a national referral hospital. On the other hand, the NTH is a referral hospital mainly serving the northern part of the country.

These two teaching hospitals’ size and pyramid-type referral systems make them ideal places to study NIs in Zambia. The two hospitals, located more than 320 km apart, were included as study sites to ascertain the diversity of AMR patterns in these two hospitals.

### Sample size estimation

According to Gosling *et al*.,^13–15^ data on the prevalence of hospital-acquired infections in Zambian hospitals were scarce. In this study, we assumed an estimated prevalence of 10% positivity to *P. aeruginosa* among patients screened for NI,^16^ a 95% confidence level of obtaining a true estimate and an allowable error of 2%. Based on these assumptions, we used the AusvetEpitool (https://epitools.ausvet.com.au/oneproportion) to estimate the sample size of 841 patients. Considering the size of the two hospitals, sample estimates were distributed proportionally according to the bed capacity. Therefore, we set to screen three quotas (640) and one quota (201) of patients at the UTH and NTH, respectively.

### Sampling

In this study, we applied consecutive sampling. We included everyone who met the inclusion criteria as they attended the hospitals (UTH and NTH) either as returning outpatients or inpatients. All consecutive (until meeting sample size) *P. aeruginosa* suspected infected patients meeting the eligibility criteria were identified and enrolled after informed consent.

### Patient selection

Participants were enrolled among adult medical and surgical patients after obtaining consent from patients or their next of kin. Hospital or NIs were defined as infections that arose during hospitalization, occurring at least 48 h after admission.^17^ The history of prior hospital contact within a month was also documented. Patients who developed clinical evidence of nosocomial wounds (surgical or pressure sore) and respiratory or urinary tract infections with a positive *P. aeruginosa* culture were included in the study.

Patients who presented with localized swelling, pain, purulent discharge, redness or heat in the skin, subcutaneous tissue, deep soft tissue, organ, or spaces and at least one positive culture for *P. aeruginosa* after 48 h post-surgical intervention were considered as nosocomial surgical site infection. Patients with fever (> 38°C), dysuria, frequency, urgency or suprapubic tenderness with no other recognized cause after 48 h of admission or recent history of catheterization were also considered. Non-catheterized patients were considered to have nosocomial urinary tract infections after obtaining a positive culture from their midstream urine.

Furthermore, patients with fever (> 38°C), chills, cough or hypotension and at least one positive culture for *P. aeruginosa* in sputum or respiratory secretions after 48 h of admission were considered to have a nosocomial respiratory infection and included as study participants. However, those patients who could not give either consent, clinical details or samples due to different conditions were excluded from the study.

### Clinical data and laboratory specimen collection

Questionnaire data were administered to enrolled patients using the Epi collect five electronic tools (https://five.epicollect.net/).^18^ Additional data were obtained from attending internists and surgeons for their decision if we could not select based on the above criteria. Face-to-face interviews were used to collect information on each patient’s socio-demographic variables and potential risk factors for NI. For those unable to provide information, the caregiver was interviewed. Clinical data on chronic diseases, hospitalization, previous admission, ward type and antimicrobial taking history were collected by reviewing the patient’s medical record and consulting the attending physician and surgeon. Clinical specimens such as urine, respiratory secretions and wound swabs were collected as soon as NI was suspected.

### Wound, urine and respiratory sample collection and processing

#### Wound sample collection

Amies media sterile swabs were used to collect discharge from patients’ septic burns, pressure sores or other wounds and at intravenous injection sites for those in a dialysis unit. Using Levine’s technique, wound/pus specimens were collected aseptically by sterile cotton swabs dipped in normal saline.^19^ This technique has been reported to detect more organisms in acute and chronic wounds than other techniques.^19,20^

### Urine sample collection

Patients suspected of non-catheterized urinary tract infection were instructed to collect 10 mL midstream clean-catch urine samples using a wide-mouth sterile container. For catheterized patients, the catheter was clamped below the port to allow for urine to collect in the tubing. The catheter was disinfected with 70% alcohol, and 10 mL of freshly voided urine was aseptically collected through the port using a syringe. The urine sample was transferred to a sterile container. While changing catheters, the medical device tips were put in normal saline and taken for cultures.

### Respiratory sample collection

In patients with chest symptoms, we collected the ‘deep cough’ sample of the early morning before eating or drinking anything to avoid bias in interpreting the results. At first, the patients needed to rinse out the mouth with clear water for 10–15 s to eliminate any contaminants in the oral cavity. After expelling saliva, the patients then breathed in deeply three times to cough at 2-min intervals until bringing up some sputum. At least 5 mL of sputum was then released in a sterile, well-closed container. ICU, the tip of the endotracheal tube was aseptically submitted for culture.

The hospital and wards of origin, date and time of collection, specimen type and tests performed were carefully noted. All specimens were placed in tightly sealed, leak-proof containers and transported in sealable, leak-proof plastic bags.

After labelling sterile containers, clinical specimens were transported to microbiology laboratories at the University of Zambia (UNZA) for UTH specimens and the Tropical Diseases Research Centre for the NTH. All samples were transported for processing within 30min to 2 h of collection.

### Processing, isolation and identification

All the specimens (swabs, urine and respiratory) were inoculated onto blood agar and MacConkey (Oxoid Ltd, Basingstoke, UK) and incubated aerobically at 37°C for 24 h. Urine was inoculated using the calibrated loop that measures about 1 μL, and after 24 h of incubation, plates were examined and inspected for bacterial growth. Colonies were counted using a colony counter and checked for significant bacteriuria. Cultures from catheterized and non-catheterized patients that grew ≥10^2^ and 10^5^ cfu/mL, respectively, were taken as considerable bacteriuria and processed further.^21^

Pure bacterial growths from wounds, urine and respiratory samples were examined for colony morphology. All flat, large and greenish non-lactose fermenting colonies on MacConkey were sub-cultured onto nutrient agar (Oxoid Ltd) and subjected to Gram staining and conventional biochemical tests for presumptive identification. Isolates that were Gram-negative rods, oxidase-positive, catalase-positive, Simon’s citrate-positive and urease-negative were considered presumptive *P. aeruginosa.*^22^

### Molecular conformation of isolates

In addition to the phenotypic analysis, molecular analysis was performed to confirm the isolates. According to the manufacturer’s instructions, DNA was extracted from purified presumptive positive cultures of *P. aeruginosa* using commercial DNA extraction kits (Qiagen, Hilden, Germany). PCR amplification was used to amplify 16S rRNA gene fragments from purified genomic DNA of culture isolates. The PCR master mix contained 1×PCR buffer [50 mM KCl, 1.5 mM MgCl_2_, 10 mM Tris/HCl (pH 9.0), 10 µg gelatine mL^−1^], 200 µM deoxynucleotide triphosphates, 0.3 µM each of the 16S rRNA primers, 1.5 U *Taq* polymerase (Gibco BRL) and ∼ 10 – 100 ng DNA in a final reaction volume of 100 µL. According to the manufacturer’s instructions, PCR based on Extaq protocol [Takara Biotechnology (Dalian) Co, Ltd] was used for species identification using specific primers targeting the 16S rRNA gene as previously described.^23,24^ PCR was also performed to identify resistant genes using specific primers for *Bla OXA 23*, *Bla OXA 51* and *Bla IMP* genes.^25^ The following thermal cycling conditions were used: 98°C for 10 s, 35 cycles of 98°C for 30 s, 54°C for 30 s and 72°C for 1 min and final extension at 72°C for 5 min. The positive amplicons were purified using Wizard SV Gel and Clean-Up System (Promega, Madison, WI, USA) according to the manufacturer’s protocol. The purified DNA was later sequenced directly using a Big Dye terminator cycle sequencing ready reaction kit v3.1 and analysed on a 3500 Genetic Analyser (Applied Biosystems, Foster City, CA, USA).

### Antimicrobial susceptibility testing

The susceptibility of *P. aeruginosa* isolates to different antibiotics was determined by Kirby–Bauer’s disk diffusion agar method on cation-adjusted Mueller–Hinton agar (Merck, Darmstadt, Germany) according to the Clinical and Laboratory Standards Institute recommendations.^26^ Antibiotic disks (MAST Diagnostics, Merseyside, UK) tested were as follows: CAZ (30 μg), TZP (100 μg/10 μg), CIP (5 μg), GEN (10 μg), AMK (30 μg), TOB (10 μg), IPM (10 μg), ATM (30 μg) and FEP (30 μg). The turbidity standard of a 0.5 McFarland (1.5 × 108 cfu/mL) of *P. aeruginosa* was prepared and spread on Mueller–Hinton agar as a lawn culture. The control strain of *P. aeruginosa (ATCC 27853)* was used to quality control antibiotic disks. *P. aeruginosa* is intrinsically resistant to CRO and CTX; therefore, *P. aeruginosa*’s sensitivity to third-generation cephalosporin was assessed based on susceptibility to CAZ.^27^

### Data analysis

The collected data were validated and entered into Excel Spreadsheets before exporting to STATA version 14.0 (College Station, TX: StataCorp LP, USA) for analysis. Descriptive statistics were computed and presented using words and tables. The OR and 95% CI were calculated to measure the strength of the association.

The frequency of NI was determined at a 95% CI. The proportions (%) of the different variables were estimated as the number of positive outcomes divided by the number examined. Quantitative variables were summarized using mean and SD. Frequency distributions of the variables were produced and checked for inconsistencies and input errors. A χ^2^ test was performed to screen potential factors associated with AMR in univariable analysis. Predictors of AMR were determined using step-wise logistic regression. The logistic regression model was tested for goodness of fit using the Hosmer*–*Lemeshow test. The level of significance was set at a *P* value of < 0.05. The nucleotide sequences obtained from the 16S rDNA analysis were compared for similarity with other previously published sequences using the BLAST search on NCBI/PubMed https://blast.ncbi.nlm.nih.gov/Blast.cgi? PAGE_TYPE=BlastSearch.

### Ethical consideration

The study protocol was ethically approved by the University of Zambia Biomedical Research Ethic Committee (UNZA REC) (ref. no. 671-2019). Participants provided informed written consent to collect anonymized clinical and demographic data. For illiterate participants, a witnessed fingerprint was obtained. All obtained information was confidential and only used for research or academic purposes. Electronic devices used to collect and store the data were password protected.

Furthermore, authorization for data collection was obtained from all legally recognized district and hospital representatives. After clarifying the study’s objective, written informed consent was obtained from adult study participants. The results were maintained anonymously and used only for the study. Positive findings were communicated to the attending clinician for appropriate treatment.

## Results

### Socio-demographic characteristics

**Figure 1.**
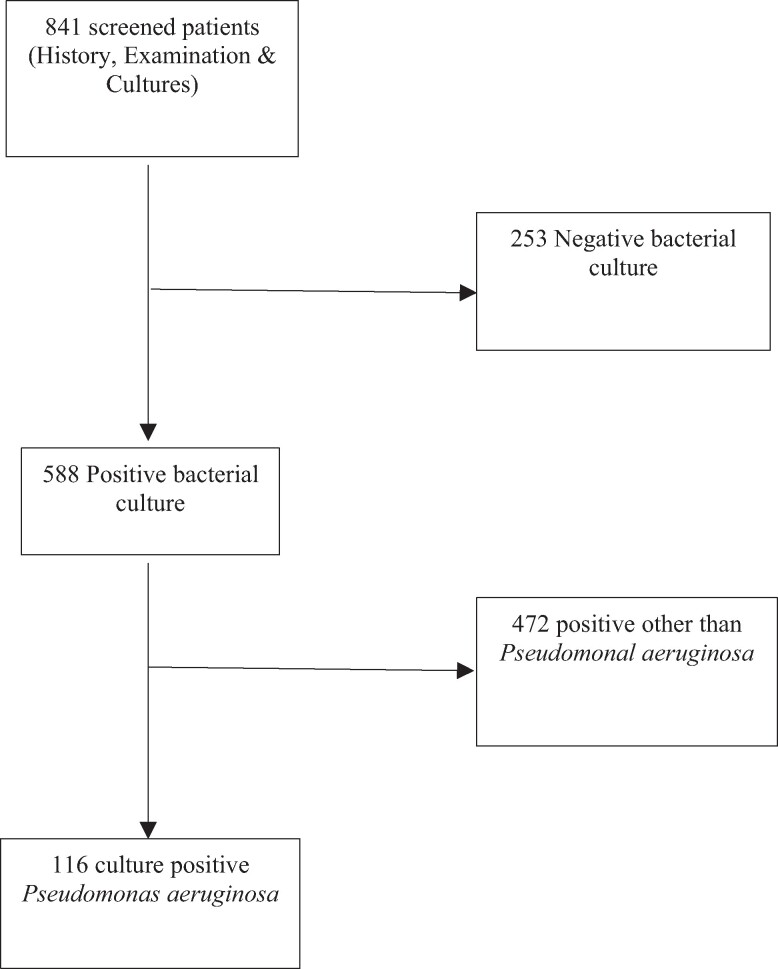
Schematic diagram of the sample processing and *P. aeruginosa* isolation.

Eight hundred and forty-one patients were screened for NIs. The majority were male (81.2%). The participants ranged from 15 to 98 years, with a mean age of 51, and most (90.4%) lived in Lusaka. Only 9.6% of participants completed tertiary and 18% (150) secondary education, while 563 achieved at least primary education. Around 6% (53) of participants reported no formal education. Only 10% (85) of participants reported being in formal employment, while the majority (756) were either in the informal sector or unemployed. Most participants (71%) were married. Of 841 screened, 588 patients (69.9%) had positive bacterial cultures (Figure 1).

The full details of these were described in a previous publication.^26^ The current study focussed on AMR of the 116 (13.7%) patients diagnosed with *P. aeruginosa* infections. Of them, 96 were male and 20 were female. Table 1 highlights the factors associated with AMR in univariable analysis.

**Table 1.** Association of clinical and molecular characteristics with AMR to *P. aeruginosa* among patients treated for NI at two tertiary hospitals, Lusaka and Copperbelt, Zambia

*Independent variables*		AMR		OR	CI	*P* value
		Yes	No			
*Gender*	Male	50	46	1.49	0.55–4.04	0.441
	Female	8	11			
*Age (years)*	15 – 65	49	50	0.84	0.35–2.00	0.826
	> 65	14	12			
*Education status*	Illiterate	27	16	2.28	1.05–5.07	0.033*
	Literate	30	41			
*Residence*	High density	36	41	0.68	0.31–1.47	0.432
	Low density	22	17			
*Occupation*	Employed	14	13	0.98	0.41–2.32	1.000
	Unemployed	48	44			
*Previous antimicrobial use*	Yes	13	17	0.73	0.31–1.69	0.522
	No	40	38			
*Hospital*	UTH	53	48	2.57	0.63–10.52	0.202
	NTH	3	7			
*Department*	Inpatient	31	27	1.37	0.65–2.88	0.453
	Outpatient	25	30			
*Wards*	Surgical	48	17	4.16	1.30–13.29	0.014*
	Medical	47	4			
*History of previous admission (30 days)*	Yes	25	33	0.86	0.39–1.86	0.843
*Prior medication*	Yes	42	46	0.66	0.24–1.80	0.458
	No	11	8			
*Invasive medical device*	Yes	3	92	0.19	0.03–1.24	0.081
	No	3	18			
*Comorbidity*	HIV+ ve	10	3	0.70	0.18–3.47	0.615
	HIV− ve	85	18			
	BPH+ve	9	10	2.25	0.78–6.45	0.165
	BPH −ve	18	45			
*Presence of resistance genes*	Amp C+ ve	43	20	0.80	0.27–2.36	0.792
	Amp C− ve	16	6			
	BLA OXA 23+ ve	7	1	3.36	0.39–28.86	0.425
	BLA OXA 23− ve	52	25			
	BLA OXA 51+ ve	65	6	5.33	1.91–16.3	0.001*
	BLA OXA 51− ve	30	15			
	BLA IMP+ ve	5	1	1.16	0.13–10.52	0.481
	BLA IMP− ve	90	21			

IPD, inpatient department; MSU, midstream urine; NTH, Ndola Teaching Hospital; OPD, outpatient Department; RTI, respiratory tract infection; UTH, University Teaching Hospital; UTI, urinary tract infection.

### Participant’s enrolment per study sites and wards

One hundred and sixteen patients were diagnosed with *P. aeruginosa* NI out of 841 screened participants from the UTH (640) and NTH (201). NI types and samples collected from patients at the UTH and NTH from different departments are shown in Table 2.

**Table 2.** NI types and samples collected from the UTH and NTH

*Hospital*	Ward	Department	UTI (MSU)	Wound (swab)	RTI (sputum)	Total
*UTH*	Medical	OPD	9	13	1	24
		IPD	14	12	2	28
	Surgical	OPD	17	12	3	32
		IPD	12	8	1	21
	Medical	OPD	0	0	0	0
*NTH*		IPD	1	3	2	6
	Surgical	IPD	1	3	0	4
		OPD	0	1	0	1
*Total*			51	56	9	116

IPD, inpatient department; MSU, midstream urine; NTH, Ndola Teaching Hospital; OPD, outpatient department; RTI, respiratory tract infection; UTH, University Teaching Hospital; UTI, urinary tract infection.

### Factors associated with *P. aeruginosa* NI

Invasive medical device-related NI was a common presentation (78.5%) among participants. However, the presence of the medical device was not associated with AMR (*P* value 0.4581). A urinary catheter was frequently inserted among surgical patients being managed for benign prostate enlargement (BPH). Catheter-associated urinary tract infection was the most common NI (57%). The other common diagnosis was infected pressure sores (38.7%). AMR was associated with carbapenem-hydrolysing β-lactamase gene *BlaOXA-51* (*P* value 0.001), literacy level (*P* value 0.033) and surgical ward attendance (*0.014).

Factors that were not associated with AMR include having an age of ≥ 65 years, male gender, prior admission and prolonged hospital admission. More than half (62%) of participants reported a history of previous hospital exposure within the past 30 days, which were prior medical visits or being ex-bed-siders taking care of patients.

### AMR profiles

The antimicrobial susceptibility and resistance profiles of all 116 clinical isolates of *P. aeruginosa* are shown in Table 3. The antibiotic sensitivity of *P. aeruginosa* was best with aminoglycosides (AMK 87%, TOB 86% and gentamycin 79%), quinolones (CIP 70%) and carbapenem (IPM 58.2%). The susceptibility to monobactam (ATM 34%), cephalosporins third-generation (ceftazidime 55.4%) and fourth-generation (FEP 3.8%) antibiotics was moderate to poor.

**Table 3.** *P. aeruginosa* antimicrobial susceptibility and resistance patterns

*Antibiotic name*	R (%)	I (%)	S (%)	R 95% CI	S 95% CI
*Piperacillin/tazobactam*	30.09	13.59	56.31	21.7–40.0	46.2–65.9
*Ceftazidime*	34.54	10	55.45	25.9–44.3	45.7–64.8
*FEP*	69.23	26.92	3.84	59.3–77.7	1.2–10.1
*Aztreonam*	39.80	25.24	34.95	30.4–49.9	26.0–45.0
*Imipenem*	6.79	34.95	58.25	3.0–14.0	48.1–67.8
*Amikacin*	2.73	10	87.27	0.7–8.4	79.2–92.6
*Gentamicin*	19.42	0.97	79.61	12.5–28.6	70.3–86.7
*Tobramycin*	8.74	4.85	86.40	4.3–16.4	77.9–92.1
*Ciprofloxacin*	24.27	4.85	70.87	16.6–33.9	61.0–79.2

I, intermediate; R, resistant; S, sensitive.

A high prevalence of multidrug resistance (MDR) (73.6%) and extensive drug resistance (XDR) (37.2%) was observed in the current study at all the two tertiary hospitals included in the present study (see Table 4).

**Table 4.** Distribution of MDR and XDR *P. aeruginosa* at the UTH and NTH

*AMR pattern*	Frequency		Total	Associated resistance genes
	UTH (*n* = 106)	NTH (*n* = 11)		
*MDR*	80 (75.5%)	5 (45.4%)	85 (73.3%)	*amp* C, *Bla OXA 23*, *bla*OXA 51
*XDR*	40 (39.6%)	1 (0.9%)	41 (37.2%)	*Amp* C, *bla*OXA 23, *bl*OXA 51, *bla*IMP

MDR, multidrug resistance: resistance to at least one agent in three or more antibiotic classes; XDR, extensive drug resistance: resistance to at least one agent in all but two or fewer antimicrobial categories.

The multivariate logistic regression model results showed that *P. aeruginosa* MDR was related to province, admission to the surgical department, current antibiotic therapy, self-medication and *bla*OXA 51 genes. Details of the logistic regression are shown in Table 5 (MDR) and Table 6 (XDR).

**Table 5.** Logistic regression analysis of the factors associated with *P. aeruginosa* NI and MDR at the UTH and NTH

*Provinces*	*0.11*	*−2.14*	*0.032*	*0.0141587*	*0.83*
*Department*	*0.15*	*−2.73*	*0.006*	*0.038*	*0.583*
*Current antibiotics treatment*	*4.36*	*2.07*	*0.039*	*1.08*	*17.59*
*blaOXA-51gene1*	*8.80*	*3.54*	*0.000*	*2.63*	*29.39*
*Self-medication*	*2.05*	*1.13*	*0.260*	*0.586*	*7.20*
*Cons*	*180.97*	*2.84*	*0.005*	*4.99*	*6561.30*

_cons estimates baseline odds.

**Table 6.** Logistic regression analysis of the factors associated with *P. aeruginosa* NI and XDR at the UTH and NTH

*Variables*	OR	*Z*	*P* > [*z*]	95% CI	
*Age group*	1.92	2.14	0.033	1.06	3.51
*blaOXA23 gene*	12.52	2.72	0.007	2.02	77.63
*blaOXA51 gene1*	4.67	3.00	0.003	1.71	12.78294
*Cons*	0.02	−3.71	0.000	0.003	0.17

_cons estimates baseline odds.

### Genomic analysis

The results of amplified genes by PCR showed that six (5.1%) *P. aeruginosa* clinical isolates contained *bla*IMP. These six clinical isolates all belonged to the UTH in Lusaka and were cultured from urinary tract infection (*n* = 4) and skin swabs from patients with infected decubitus ulcers (*n* = 2). All these six patients were from the surgical outpatient department (50%) and the medical outpatient department (50%) with a history of prior medical contact within 90 days and self-medication. These clinical isolates were MDR and harboured *amp* C and *bla*OXA51 genes. Table 7 highlights the frequency of targeted resistance genes.

**Table 7.** *P. aeruginosa* analysis of targeted resistance genes

*Genomic analysis*				
*Genes and primers used*		+ve	−ve	%
*Primers*	*PA-SS*	87	25	112 (96%)
	GGGGGATCTTCGGACCTCA (F)			
	TCCTTAGAGTGCCCACCCG (R)			
	*PA-GS*	70	16	86 (74%)
	GACGGGTGAGTAATGCCTA (F)			
	CACTGGTGTTCCTTCCTATA (R)			
	*AMP C*	79	20	99 (85.3%)
	CGGCTCGGTGAGCAAGACCTTC (F)			
	AGTCGCGGATCTGTGCCTGGTC (R)			
	*Bla Oxa23*	7	1	8 (6%)
	GATCGGATTGGAGAACCAGA (F)			
	ATTTCTGACCGCATTTCCAT (R)			
	*Bla Oxa51*	65	7	72 (62%)
	TAATGCTTTGATCGGCCTTG (F)			
	TGGATTGCACTTCATCTTGG (R)			
	*Bla IMP*	6	0	6 (5%)
	GTTTGAAGAAGTTAACGGGTGG (F)			
	ATAATTTGGCGGACTTTGGC (R)			

**Figure 2.**
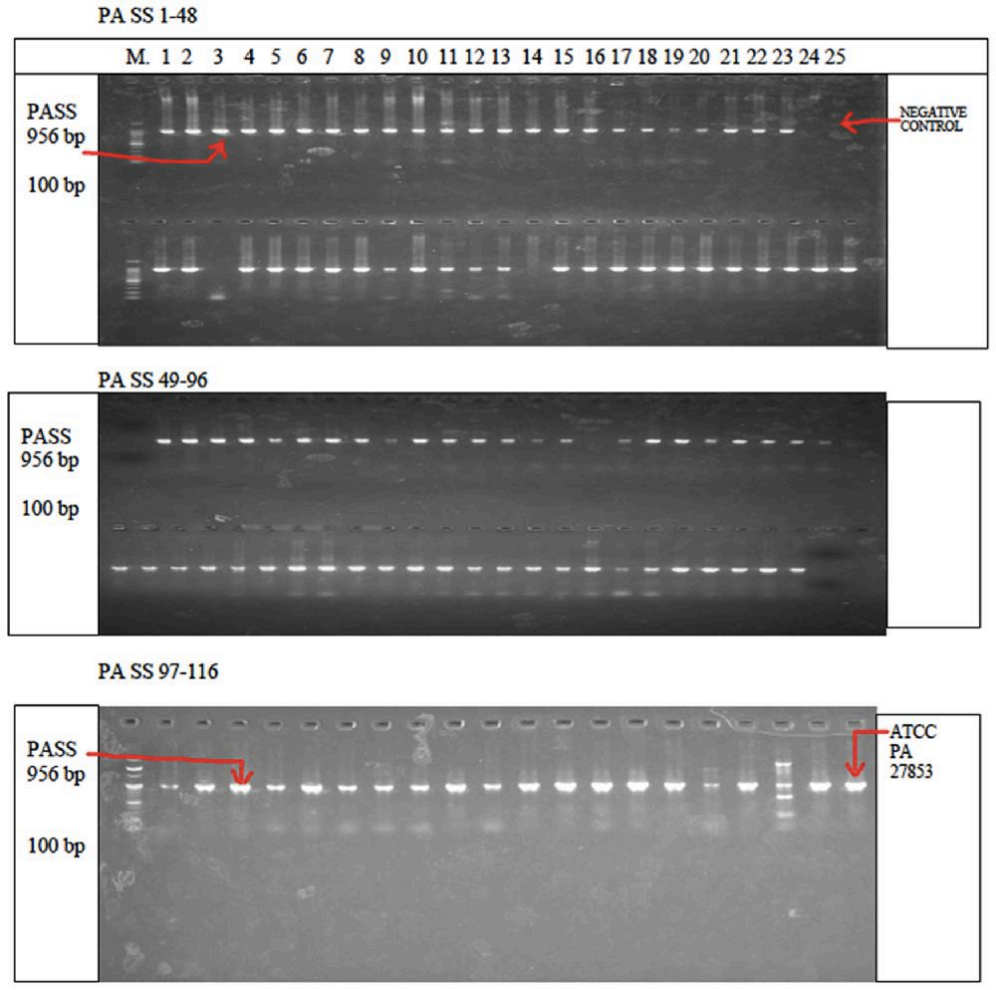
An electrophoretogram showed amplicons of 116 clinical isolates of *P. aeruginosa* using specific primers (*PA*-*SS*). This figure contains clinical isolates from 1 to 48, 49 to 96 and 97 to 116. PA-SS-positive amplicons are shown in lanes 1–23, 26, 28–37, 39–48, 49–63, 65–72, 73–96 and 97–115. PA-SS-negative amplicons are shown in lanes 24, 27, 38 and 64. Ladder (lane M): 100 bp molecular maker, P: positive control, N: negative control, *25*. Positive control *P. aeruginosa* ATCC 27853 (116) (New England Biolabs Inc.).

**Figure 3.**
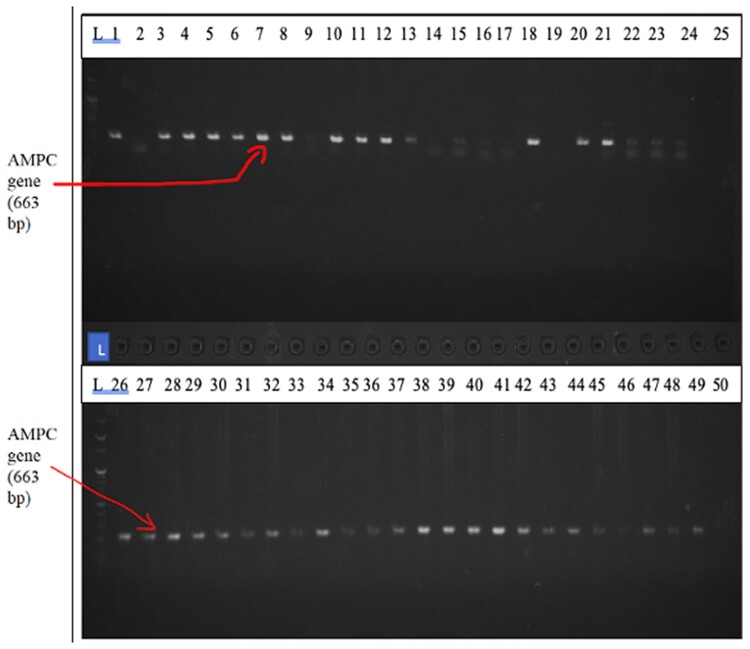
Agarose gel electrophoresis of PCR product for the presence (1, 3, 4, 5, 6, 7, 8, 10, 11, 12, 13, 18, 20–49) or absence (2, 9, 14–17, 19) of *amp* C gene. A negative control is shown in well 50. Lane L represents the 100 pb DNA ladder.

**Figure 4.**
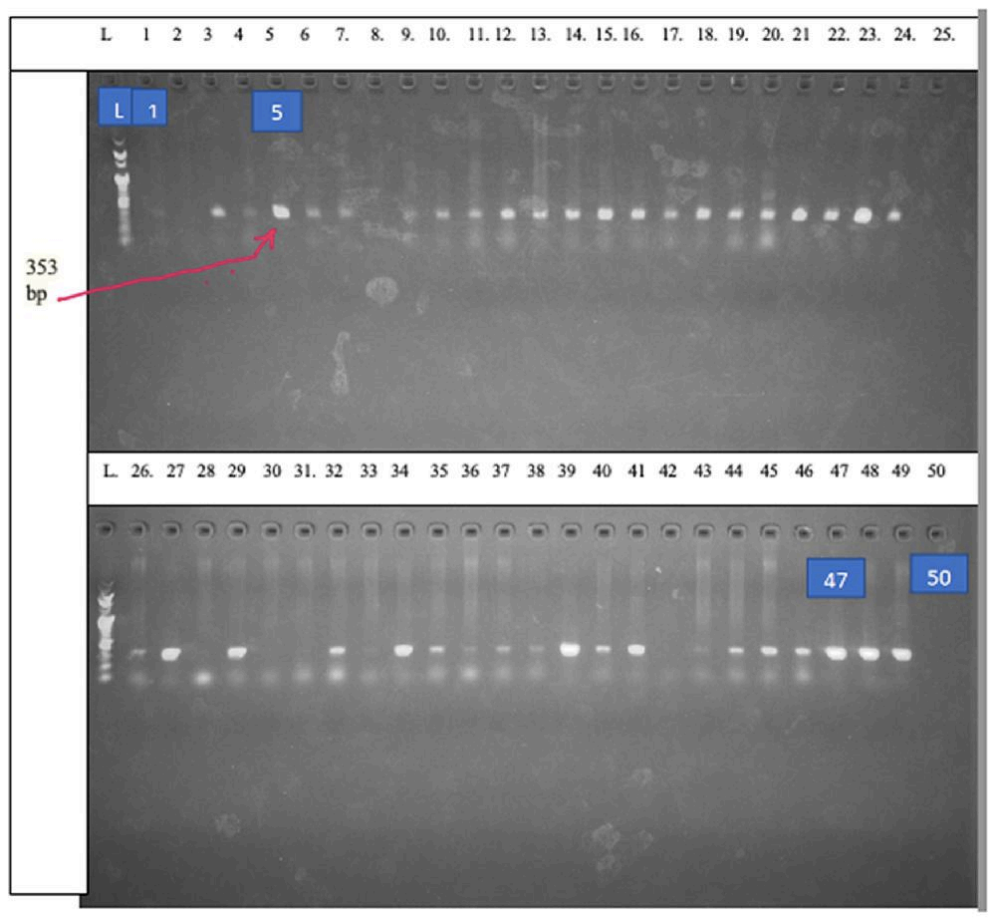
Agarose gel electrophoresis of the PCR product for the presence (3, 4, 5, 6, 9–24, 26, 29, 32, 34, 35, 37, 38, 39, 40, 41, 44–49) or absence (1, 2, 4, 8, 9, 25, 28, 30, 31, 33, 42 and 43) of the *blaOXA51* gene. Wells 25 and 50 show a negative control. Lane L represents the 100 pb DNA ladder.

The results of a PCR assay for 116 clinical isolates showed that the majority 99/116 (85.3%) of *P. aeruginosa* clinical isolates harboured *the amp* C *gene.* These 99 clinical isolates were recovered from the UTH (90) and NTH (9) in patients presenting with urinary tract infection (49) (49.5%), infected wounds (37) (37.4%) and respiratory tract infection (5%). Figures 2–4 show agarose gel electrophoresis of the PCR product to determine the presence of PA-SS, *amp* C and *bla*OXA51 genes in *P. aeruginosa* clinical isolates.

The results of amplified genes by PCR showed that only eight (6%) clinical isolates contained *bla*OXA23. At the NTH, *bla*OXA23 was found in 10% of isolates from infected wounds and 6% of isolates from the UTH from patients with infected wounds (*n* = 2), urinary infection (*n* = 3) and respiratory tract infection (*n* = 2).

The results of amplified genes by PCR also showed that 72 (62%) clinical isolates contained *bla*OXA51. At the NTH, 40% of *P. aeruginosa* recovered from infected wounds (*n* = 4) harboured *bla*OXA51. At the UTH in Lusaka, 64.1% of *P. aeruginosa* recovered from infected wounds (*n* = 21), urinary tract infection (*n* = 40) and respiratory tract infection (*n* = 5) harboured *the bla*OXA51 gene.

## Discussion

NIs caused by antibiotic-resistant *P. aeruginosa* have emerged as a primary concern in clinical care settings due to the increasing development of MDR strains.^6^ This study evaluated AMR in patients in two tertiary hospitals in Zambia. The majority of participants were male patients with indwelling urinary catheters. In a study conducted in Ethiopia, Taye *et al*.^28^ reported a 4.62 higher risk of developing NIs in patients with urinary catheters than non-catheterized chronic patients. This observation is consistent with other studies done in Poland,^29^ China,^30^ India,^31^ Morocco^32^ and Egypt.^33^

In the current study, most participants were enrolled from the UTH compared with the NTH. At the NTH, the data were collected during the pick of the COVID-19 third wave, hence the low enrolment (23.9%) of participants at this site due to a decrease in the number of patients attending hospital for non-COVID 19-related illnesses during the study period. Similarly, Quadros *et al*.^34^ linked the reduction of patients attending hospitals with non-COVID-19-related diseases during an outbreak to public anxiety about acquiring the viral infection in the hospital and the subsequent risk of mortality and lockdown.

The current study found self-medication to be associated with AMR. Similarly, Miliani *et al*.^35^ reported that AMR was associated with the common usage of antibiotics. Likewise, Leopold *et al*.^36^ and Tadesse *et al*.^37^ reported a high resistance level to commonly used antibiotics compared with less prescribed antibiotics in sub-Saharan Africa. Despite the development by the WHO of the Access, Watch and Reserve classification system to guide the use of antibiotics and prevent AMR,^38^ there are numerous challenges in the implementation in various countries due to a lack of awareness, limited resources and capacity to implement the framework in many countries, especially in lower- and middle-income countries, including Zambia. This could be due to the low availability of affordable and good-quality antibiotics, leading to the inappropriate use of antibiotics and AMR.^39^ The association between self-medication and AMR observed in our study could also result from repeated exposure to antimicrobial agents through over-the-counter access to these drugs.^40,41^

The current study found high resistance levels to CAZ and ATM despite the report that *P. aeruginosa* is naturally susceptible to carboxypenicillins, ceftazidime and aztreonam.^6^ This could be explained by the high prevalence of Amp C β-lactamase in the current study.

The present study also observed a high resistance level to piperacillin despite being combined with a β-lactamase inhibitor (tazobactam). This could also result from the increased expression of the Amp C gene observed in our clinical isolates of *P. aeruginosa*. Amp C cephalosporinase activity is not inhibited by β-lactamase inhibitors used in clinical practice, such as CLA, SUL and TZB.

Resistance of *P. aeruginosa* to CIP is a rising problem in many parts of the world. In the current study, the resistance rate to CIP was low but higher than that reported in some countries in the Middle East.^42^ This was similar to the findings by Yang *et al*.^42^ who reported low AK and CIP resistance among *P. aeruginosa* in South China. Due to the relatively low resistance of *P. aeruginosa* to fluoroquinolones among our clinical isolates, the mutation of *gyrA*/*gyrB* genes within the quinolone-resistance-determining region linked to fluoroquinolones resistance of *P. aeruginosa* was not investigated.

The multivariate logistic regression model in the present study showed that *P. aeruginosa* AMR was associated with self-medication, surgical OPD attendance or ward admission and the carbapenem-hydrolysing β-lactamases gene *blaOXA-51*. Generally, self-medication has been reported to be associated with emergency of AMR in bacteria,^43,44^ and thus, this practice needs public sensitization. In a related study, Yamba *et al*.^45^ observed high morbidity and mortality rates associated with AMR pathogens in the same hospital (UTH) surgical ward, indicating the need to improve infection prevention and control in the surgical wards.

### Conclusion

This study has demonstrated that multidrug-resistant *P. aeruginosa* is highly prevalent in hospitals in Zambia’s Lusaka and Ndola districts and possibly countrywide. *P. aeruginosa* AMR was associated with self-medication, surgical care and carbapenem-hydrolysing β-lactamase gene *blaOXA-51*. This calls for the establishment and implementation of antimicrobial stewardship programmes and the strengthening of the surveillance system.

## Limitations

The study was conducted at two large teaching hospitals, though including data from the regional hospital could give an idea of the actual burden of *P. aeruginosa* AMR countrywide. This study should expand to regional hospitals to obtain the real-burden AMR profiles.
